# De novo mitochondrial genome sequencing of *Cladonia subulata* and phylogenetic analysis with other dissimilar species

**DOI:** 10.1371/journal.pone.0285818

**Published:** 2023-05-23

**Authors:** Jinjin Fang, Reyim Mamut, Lidan Wang, Gulmira Anwar

**Affiliations:** College of Life Science and Technology, Xinjiang University, Urumqi, China; ICAR Research Complex for Eastern Region, INDIA

## Abstract

In this study, the complete mitochondrial genome of *Cladonia subulata* (L.) FH Wigg was sequenced and assembled and then compared with those of other *Cladonia* species. The mitogenome of *Cladonia subulata*, the type species of *Cladonia*, consisted of a circular DNA molecule of 58,895 bp 44 genes (15 protein-coding genes, 2 rRNA genes, and 27 tRNA genes). The base composition had shown an obvious AT preference, and all 27 tRNA genes formed a typical clover structure. Comparison with other 7 *Cladonia* species indicated that the duplication/loss of tRNAs had occurred during evolution, and introns appeared to explain the variation in *cox*1 genes in *Cladonia*, the mitochondrial genome tends to be generally conservative and local dynamic changes. Repeat sequences were mainly located in gene intervals, which were mainly distributed among intergenic spacers and may cause rearrangement of the mitogenome. The phylogenetic results showed that *Cladonia subulata* and *C*. *polycarpoides* were assigned to the *Cladonia* Subclade. The results add to the available mitochondrial genome sequence information of *Cladonia subulata*, provide basic data for the systematic development, resource protection, and genetic diversity research in *Cladonia subulata*, and also provide theoretical support for further genomic research of lichens.

## Introduction

Mitochondria play an important role in eukaryotes by producing adenosine triphosphate (ATP) through oxidative phosphorylation, and mitochondria contain their own genome. In contrast to the nuclear genome, the mitochondrial genome usually had its own coding system and follows the rules of uniparental inheritance, which is not affected by the recombination of two parental chromosomes [1]. Because of its rapid evolution and high copy number per cell, mitogenome DNA has been widely used to study the origin, evolution, and phylogeny of fungi [2–4]. The fungal mitogenome DNA shows a high DNA AT-content and a wide range of genome sizes [5]. The fungal mitogenome contains 14 protein-coding genes (PCGs) (*nad*1-6, *nad*4L, *cob*, *cox*1-3, *atp*6, *atp*8 and *atp*9) that are related to electron transport and oxidative phosphorylation in the inner membrane of mitochondria. It also includes the mitochondrial 16S ribosomal small subunit RNA gene (*rrns*), the mitochondrial 23S ribosomal large subunit RNA gene (*rrnl*) and a variety of tRNA genes necessary for translation [6].

Lichens, are pioneer colonizers, that play a major role in studies aimed reconstructing early life on land [7–10]. A recent study suggests that lichens are self-sustaining ecosystems main consisting of Ascomycota or Basidiomycota fungi and prokaryotic or eukaryotic algae, and other microscopic organisms [11,12]. This special symbiotic system allows lichens to survive in extreme environments such as waterless deserts or areas of extreme cold [13]. In addition, lichens also play an extremely important role in antibacterial and antitumor activities because of their rich lichenic acid [14–19].

Lichens show rich morphological and ecological diversity in nature [20], however, the evidence based on morphological and chemical characteristics is not enough to accurately reflect the species diversity or species delimitation, and may misrepresent the diversity of lichenized fungi, especially in closely related species that have very similar morphological, the combination of molecular data can more accurately classify species [21–23]. Mitogenome is not only conducive to solving the problems of taxonomic identification, especially some symbiotes (such as arbuscular mycorrhizal fungi) that are difficult to be characterized by conventional methods [24], but also conducive to the comparative analysis of mitogenome to reveal the evolutionary events between species, such as the dynamic changes of genes [25].

*Cladonia* is one of the largest genera of lichens with approximately 475 species showing a variety of morphologies and habits [26], and this genus has been used in ecological research as well as in research related to industry and medicine [27–30]. To date (2023.02.01), almost 17 complete mitogenomes of this genus have been released in the NCBI database, among which the complete mitogenome sizes from 46.2 to 66.1 kb, and few of their mitogenes have been annotated or analysed, the available mitogenome data for *Cladonia* are far less abundant than those for nuclear genomes (https://www.ncbi.nlm.nih.gov/nuccore), so there are still some gaps in *Cladonia* phylogenetics and comparative genomics.

*Cladonia subulata* is the type species of *Cladonia* [31], and has been included in the "Chinese Biodiversity Red List—Macrofungi" published by the Chinese Academy of Sciences, Ministry of Ecology and Environment, as a species of LC grade (www.mee.gov.cn). In a recent study, it was found that *Cladonia subulata* showed strong cytotoxic activity which could provide a potential treatment for cancer [19]. *Cladonia subulata* has many morphological characters and can be distinguished based on lichen acids, podetia, and internal transcribed spacer sequences (ITS) [32]. Compared with the simple nuclear genome fragment, the complete mitochondrial genome could provide more abundant genetic resources for these protected species and conducive to the exploration of its functional genes [33]. The composition, repeat sequence, gene arrangement and other differences in mitogenomes could reflect the genetic evolution level in species [34], which is beneficial to the protection of the diversity of biological genetic resources and the study of species population structure by mitogenomes [24,35]. However, all of nucleotide records of *Cladonia subulata* in NCBI commonly used in phylogenetic and/or molecular systematic studies, the research on the mitogenome of *C*. *subulata* is still lacking. This information gap limits our understanding of *Cladonia subulata* at a genetic level.

In this study, we first assembled and annotated the complete mitogenome of *Cladonia subulata*. To reveal the characteristics of its mitogenome, and its differences from those of other species in the genus, the mitogenomes of *Cladonia subulata* and seven other species in the genus were compared.

## Materials and methods

### Sampling, DNA extraction, PCR and sequencing

*Cladonia subulata* was collected from Urumqi No.1 Glacier in the Tianshan Mountains of Xinjiang, China (43°13′30″ N, 87°9′11″ E), and the collection confirmed to the law of ‘the People’s Republic of China on the protection of wildlife’. The voucher specimen *Cladonia subulata* (YH0097) was deposited in the “*Lichen’s Research Center in Arid Zones of Northwest China*”, Xinjiang University, Xinjiang, China. We first identified this specimen based on morphology [20,32]: Squamules greyish green to green, esorediate or sometimes whitish sorediate at the margin. Potedia abundant, 15–40 mm tall, usually unbranched, greyish green, subulate but finally forming cup at the tip. Surface ecorticate, covered by powdery soredia, and small podetial squamules occurring at the base. Apothecia seldom, stalked, brown. Spot tests: K-, C-, KC-, P +red. Secondary metabolite: fumarprotocetraric acid (S1 Fig).

Total genomic DNA was extracted using the Fungi Genomic DNA Extraction Kit (Solarbio, Beijing, China) and stored at -20°C. Then, rDNA-ITS, mtSSU and *RPB*2 were selected as the gene markers via PCR, rDNA-ITS was used a pair of primers ITS1F [36] and ITS4 [37]; for mtSSU the primer pair mtSSUl and mtSSU3R [38]; the *RPB*2 was amplified using nested PCR [34], the primers RPB2-5F and RPB2-7R [39] were used firstly, RPB2dRaq and RPB2rRaq [32] was used for second reaction. And their conditions of PCR were following the S1 Table. Then, the PCR products were sequenced by Sangon Biotech Co. Ltd. (Shanghai, China). The ITS, mtSSU and *RPB*2 sequences were submitted to NCBI under accession numbers ON920702, ON937425 and OQ025089. After sample QC, the gDNA was used to construct a single-stranded circular (ssCir) library, and the ssCir library was then amplified through rolling circle amplification (RCA) to obtain DNA nanoballs (DNBs), which were loaded into flow cells and sequenced on the DNBSEQ Platform by BGI Biotechnology Co. (Shenzhen, China). Approximately 6 Gb of raw data were generated for each library, and the total read Q30 was over 80%.

### De novo assembly and annotation of the mitogenome

The mitogenome was extracted and assembled de novo from the whole genome dataset using GetOrganelle v1.7.5, which employs implemented SPAdes v3.13.0 assembly program [40]. The best results were obtained with K-mer = 95, and the mitogenome was represented as a circular sequence. Mitogenome annotation was performed using methods proposed by Yildiz [41]. The mitogenome was automatically annotated using the GeSeq (https://chlorobox.mpimp-golm.mpg.de/geseq.html) [42] tool based on the mould mitogenome genetic code, ORFfinder (https://www.ncbi.nlm.nih.gov/orfnder/) was used to annotate protein-encoding genes, tRNA was annotated by tRNAscan-SE v2.0 [43] and ARWEN v1.2 [44], start and stop codons were manually modified by Geneious v2022.0.1 [45]. The *Cladonia subulata* mitogenome map was drawn by OGDRAW (https://chlorobox.mpimp-golm.mpg.de/OGDraw.html) [46].

### Sequence analysis

The complete mitogenome sequence of *Cladonia subulata* and corresponding gene annotations were deposited in the GenBank database under accession number of ON882032. The predicted secondary structures of the tRNA genes were plotted by using the MITOS Web Server [47,48]. Composition skew values were calculated according to the following formulas: AT-skew = A−T/A+T and GC skew = G−C/G+C [49]. Codon usage was analysed and visualized using MEGA v7.0. Subsequently, simple sequence repeats (SSRs) were detected using MISA Perl Script (http://pgrc.ipk-gatersleben.de/misa/) [50], and tandem repeats were analysed using Tandem Repeats Finder (https://tandem.bu.edu/trf/trf.html) [51]. Repeated sequences were identified by REPuter (https://bibiserv.cebitec.uni-bielefeld.de/reputer) [52]. Genome rearrangements were aligned with progressive Mauve v2.4.0 [53].

### Phylogenetic analysis

To further prove from a molecular point of view that the species was *Cladonia subulata*. The ITS, mtSSU and *RPB*2 sequences were concatenated to verify *C*. *subulata*, *Pseudevernia cladonia* as an outgroup and three loci in *Cladonia* were extracted from GeneBank (S2 Table) followed by BLASTN with corresponding *C*. *subulata* genes using MAFFT [54], Gblocks was used to remove ambiguous regions and keep conserved regions of the sequences. Best-fit model according to Bayesian Information Criterion (BIC) by ModelFinder [55] which were used to the MrBayes: TIM3e+G4: ITS+*RPB*2; K3Pu+F+G4: SSU. Bayesian analyses were performed using MrBayes v.3.1.2 [56] with the following main parameters: ngen = 1000000, nchains = 4, simple freq = 100, nst = 6, rates = gamma, burn in = 0.25. The tree was viewed in FigTree v1.4.3 [57]. The results showed that our species and *Cladonia subulata* were included in the same clade (S2 Fig).

The complete mitogenomes of *Cladonia subulata* and 23 Lecanorales species from GenBank (S3 Table) were used for phylogenetic comparison. *Heterodermia casarettiana* (A. Massal.) Trevis. (NC 042185) and *H*. *speciosa* (Wulfen) Trevis. (NC 040159) were used as outgroup taxa [58,59]. The phylogenetic analysis used 15 PCGs of mitogenomes, which were first aligned using MAFFT. After editing and trimming to produce a sequence matrix, the results were concatenated by PhyloSuite [60]. ModelFinder [56] was used to select the best-fit partition model of each PCG (edge-linked) for maximum likelihood (ML) and Bayesian analyses by using the AIC (S4 Table). An ML analysis was performed using IQ-tree [61] incorporated in PhyloSuite under the Edge-linked partition model with 5000 ultrafast bootstraps replicates. Subsequently, Bayesian analyses were conducted using the Markov chain Monte Carlo (MCMC) method in MrBayes v.3.1.2 according to the Kundu [62] with the following conditions: the partition model was chosen (S4 Table), then the tree was then run for 10,000,000 generations with 25% burn in, with trees saved saving at every 100 generations. MCMC analysis was used to generate the convergence metrics until the standard deviation (SD) of split frequencies fell below 0.01 and the potential scale reduction factor (PSRF) for all parameters approached 1.0. Both BI and ML phylogenetic trees were viewed and edited via the web based iTOL tool (https://itol.embl.de/) [63].

## Results and discussion

### Mitogenomic characterization of Cladonia subulata

**Fig 1:**
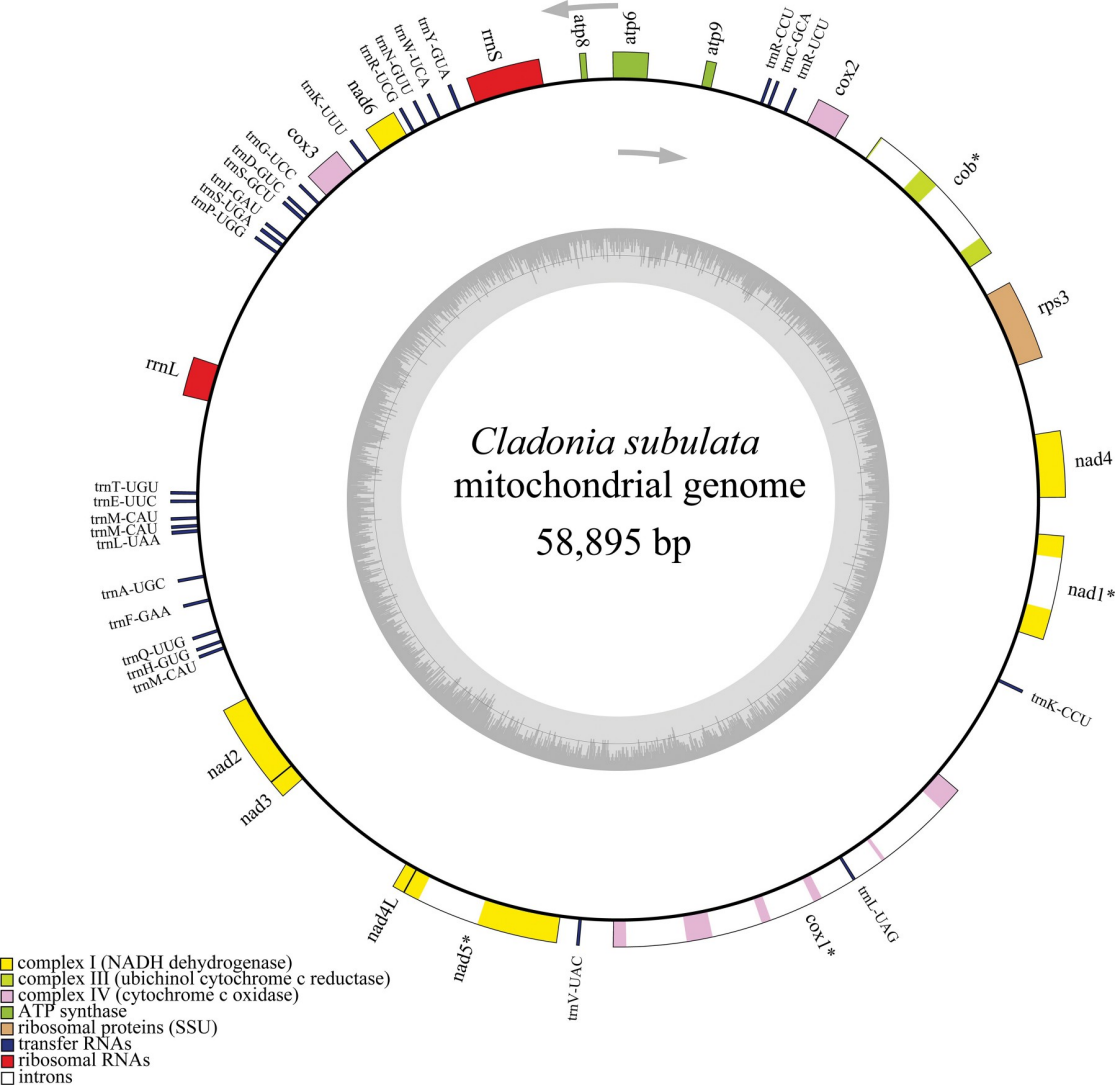
Circular maps of the mitochondrial genome of *Cladonia subulata*.

The complete mitogenome of *Cladonia subulata* was assembled as a circular molecule of 58,895 bp (Fig 1), including 15 PCGs, seven for NAD dehydrogenases (*nad*1, *nad*2, *nad*3, *nad*4, *nad*4L, *nad*5, *nad*6), three for cytochrome oxidases (*cox*1, *cox*2, *cox*3), three for ATP synthases (*atp*6, *atp*8, *atp*9), one for cytochrome b (*cob*) and one for ribosomal protein subunit 3 (*rps*3), 2 ribosomal RNA genes (*rrnS*, *rrnL*) and 27 transfer RNA genes (Table 1). All genes were transcribed at the same strand. The length of 15 PCGs varied from 147 bp (*atp*8) to 8,208 (*cox*1) and the total length was 26,194 bp which accounted for 44.48% of the mitogenome. The length of tRNAs was 1989 bp, being 3.38% of the mitogenome, while rRNAs was 2,420 bp, which was 4.11% of the mitogenome. Previous study [64] has indicated that the intron could influence the size of lichenized fungi. In our study, the mitogenome contained 11,583 bp introns in PCGs, and the introns accounted for 44.22% of the PCGs and 19.67% of the mitogenome, especially in *cox*1, which contained 79.57% introns.

**Table 1:** PCGs and rRNA gene composition of *Cladonia subulata*.

Gene	Location	start/stop codon	Introns numbers	Introns length	Exon length	Strand	Coding sequence percentage(%)	AT-skew	GC-skew
**/**	1–58895	/	9	11583	/	/	/	-0.015	0.068
***nad*4**	39–1457	GTG/TAA	0	0	1419	+	100	-0.194	0.027
***rps*3**	3028–4749	TTA/TAG	0	0	1722	+	100	0.098	0.004
***cob***	5463–9124	ATA/TAG	2	2535	843	+	33.24	-0.008	0.062
***cox*2**	9647–10372	GTG/TAA	0	0	726	+	100	-0.060	0.139
***atp*9**	12636–12860	ATG/TAA	0	0	225	+	100	-0.182	0.295
***atp*6**	14073–14840	ATG/TAG	0	0	768	+	100	-0.140	0.063
***atp*8**	15397–15543	ATG/TAA	0	0	147	+	100	-0.162	-0.238
***rrnS***	16387–17967	/	0	0	1581	+	100	/	/
***nad*6**	19645–20337	ATG/TAA	0	0	693	+	100	-0.111	0.144
***cox*3**	21090–21899	ATG/TAA	0	0	810	+	100	-0.211	0.056
***rrnL***	26408–27246	/	0	0	839	+	100	/	/
***nad*2**	34042–35853	ATG/TAA	0	0	1812	+	100	-0.136	-0.022
***nad*3**	35854–36243	ATG/TAA	0	0	390	+	100	-0.209	0.161
***nad*4L**	39222–39491	ATG/TAA	0	0	270	+	100	0.020	0.189
***nad*5**	39491–42882	ATG/TAA	1	1373	2019	+	59.54	-0.077	0.127
***cox*1**	44061–52268	ATG/TAA	5	6531	1677	+	20.43	0.045	0.086
***nad*1**	55903–58135	ATG/TAG	1	1144	1089	+	48.79	-0.021	0.052
**PCGs**	/	/	9	11583	14611	+	55.78	-0.030	0.074

The GC content of mitogenomes varies among different species, which may be affected by mutation bias, selection and recombination-related DNA repair bias [65]. The nucleotide composition of *Cladonia subulata* showed an AT bias (70.70%) and the GC content was 29.30%, similar to most *Cladonia* species (Tables 1 and 2). According to the second parity rule of Chargaff (A-T and G-C within one stand) [66], the mitogenome of *Cladonia subulata* presented a negative AT skew (-0.015) and a positive GC skew (0.068) (Table 1). This indicates that the mitogenome of *Cladonia subulata* contains more T than A nucleotides and more G than C nucleotides, as reported in other mitogenomes of *Cladonia*.

**Table 2:** Comparison of *Cladonia* mitogenomes.

Species		*Cladonia peziziformis*	*Cladonia macilenta*	*Cladonia leporina*	*Cladonia apodocarpa*	*Cladonia petrophila*	*Cladonia subulata*	*Cladonia rangiferina*	*Cladonia subtenuis*
**Length (bp)**		45312	46553	50045	50682	53100	**58895**	59116	59878
**GC content (%)**		29.70%	29.00%	29.10%	29.30%	29.30%	**29.30%**	29.60%	29.50%
**PCGs**									
	Number	15	15	15	15	15	**15**	15	16
	Length (bp)	17855	18776	23222	20016	24762	**26194**	28247	28432
	Ratio (%)	39.40	40.33	46.40	39.49	46.63	44.48	47.78	47.48
**tRNAs**									
	Number	25	19	28	22	25	**27**	23	26
	Length (bp)	1860	1415	2105	1645	1874	**1989**	1705	1952
	Ratio (%)	4.10	3.04	4.21	3.25	3.53	**3.38**	2.88	3.26
**rRNAs**									
	Number	2	2	2	2	2	**2**	2	2
	Length (bp)	4682	5157	3997	4783	4780	**2420**	3428	4735
	Ratio (%)	10.33	11.08	7.99	9.44	9.00	**4.11**	5.80	7.91
**Introns**									
	Number	4	3	7	6	10	**9**	17	12
	Length (bp)	2617	2938	7503	4801	9070	**11583**	13642	12035
	Ratio (%)	5.78	6.31	14.99	9.47	17.08	19.67	23.08	20.10

Four PCGs (*nad*4, *rps*3, *cob*, and *cox*2) were initiated with GTG, TTA, ATA, and GTG respectively. Other PCGs were started with the typical ATG start codons. The majority of PCGs were terminated by typical TAA codons, except for *rps*3, *cob*, *atp*6 and *nad*1 which had terminal codons TAG (Table 1). In addition, the first nucleotide (A) of the *nad*5 initiation codon ATG was also the last nucleotide of the *nad*4L termination codon TAA (Tables 1 and S5). All protein and RNA genes were encoded on the positive strand, the *trn*L was included in *cox*1 (Fig 1, S5 Table). All rRNAs were independent, and the longest distance was 1,581 (*rrnS*). Subsequently, 16 tRNAs located both sides of *rrnS* (S5 Table). This phenomenon suggests that the mitogenome of *Cladonia subulata* has a non-continuous segment and lack of overlapping genes (S5 Table).

**Fig 2:**
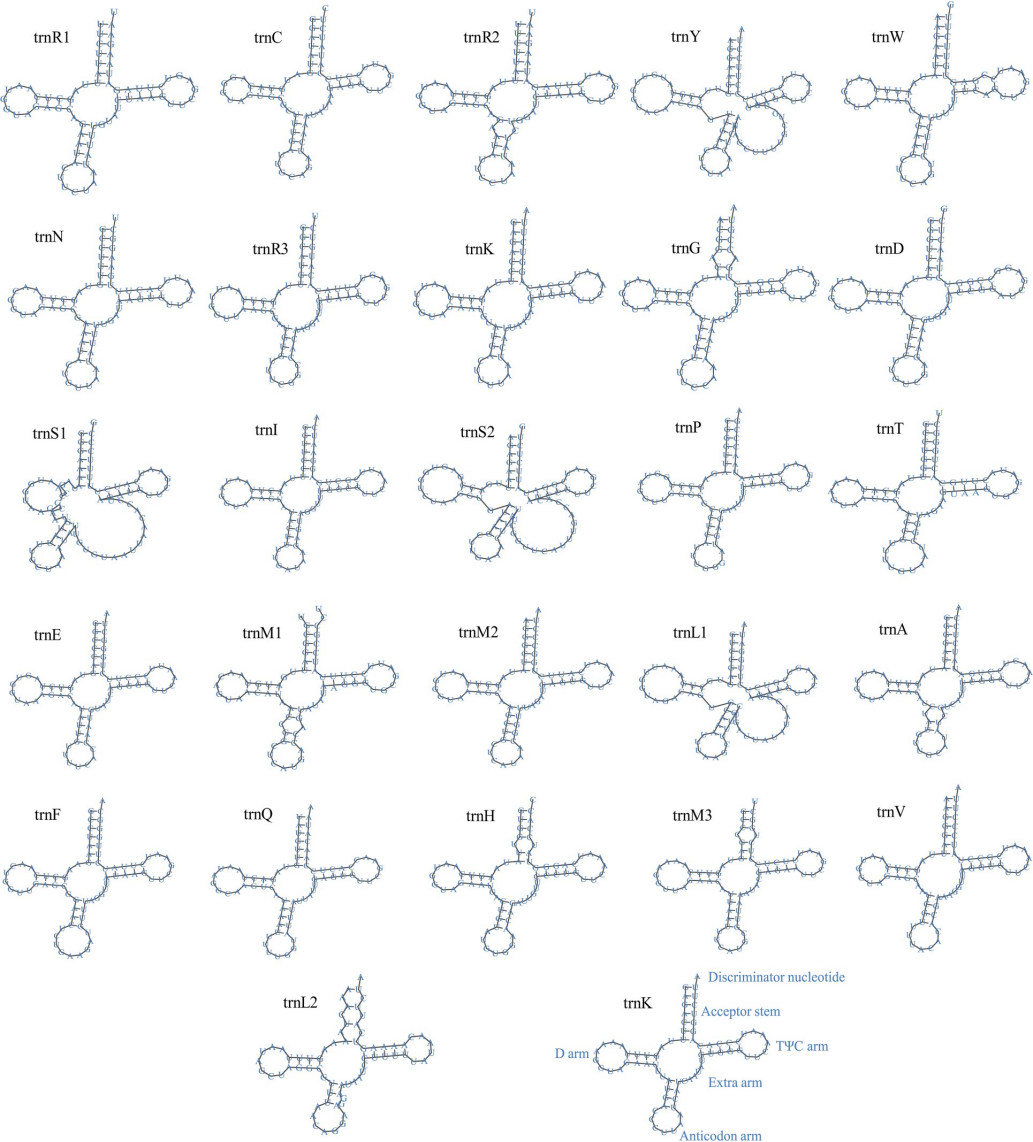
Predicted secondary structure of the 27 typical tRNA genes in the *Cladonia subulata* mitogenome.

tRNAs present the same significance during translation as mRNAs and proteins [67]. In *Cladonia subulata*, 27 tRNA genes in the mitogenome encoded the 20 standard amino acids, and the secondary structures of all tRNAs were successfully predicted ranging from 69 bp to 86 bp (Fig 2, S5 Table). Different types of analyses may result in differences in the predicted typical tRNA secondary structure. In addition, 27 tRNAs exhibited typical clover-leaf secondary structure; e.g., *trn*Y (Tyr), *trn*S (Ser), and *trn*L (Leu) showed a variable loop. All tRNAs presented typical clover-leaf secondary structures, and the remaining mismatched bases had showed non-canonical G-U pairs (Fig 2). In accordance with the analysis of secondary structure in fungi, the variations in the numbers of extra arms may cause differences in tRNA length.

**Fig 3:**
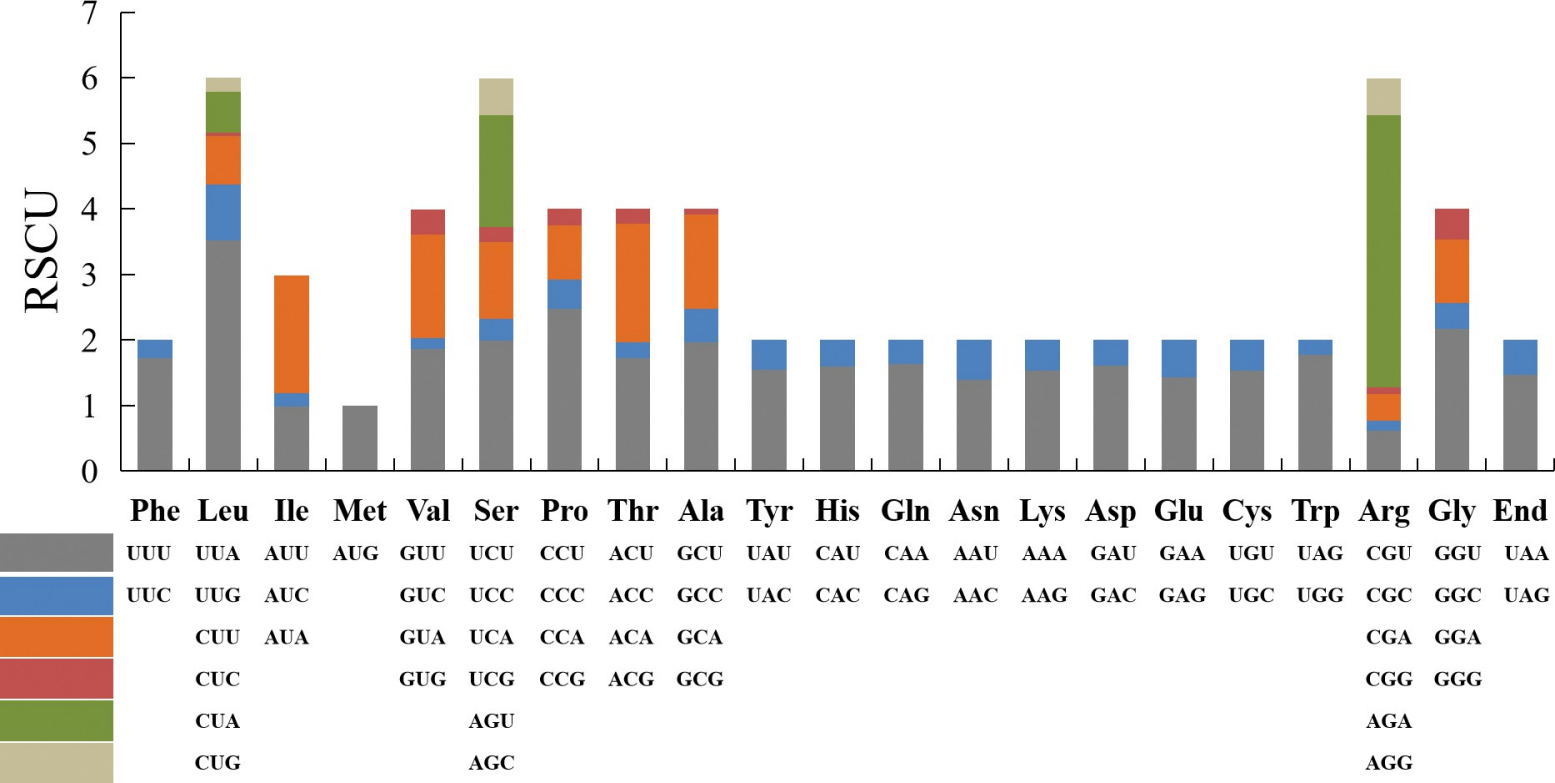
Codon usage in the *Cladonia subulata* mitochondrial genome. Codon families are indicated below the X-axis. The frequency of codon usage is plotted on the Y-axis.

Codons are carriers used for identifying and transmitting the genetic information of organisms and play an important role in biological genetics and variation [68]. The codon usage patterns of fungal mitochondria are generally similar, including the most commonly used codons UUU, UUA, AUU, AUG, GUU, CUU, AUA, and GUA, and 30 other codons, with exception of UAA. According to the analysis of codon usage, the most frequently used codons were AGA (for Arg), UUA (for Leu), CCU (for Pro). It is observed that the frequency of A and U in the codon is very high (Fig 3, S6 Table).

### Comparative mitogenomes of Cladonia fungi

The seven published and annotated fungal mitogenomes of *Cladonia* were compared to determine the variation in fungal mitogenomes. In these 8 species, the mitogenome size from 45,312 to 59,878 bp. The length of PCGs was the main reason for the differences in mitogenome length (Table 2). The tRNAs number various from 19 to 27, which indicated that some species may have lost genes in the course of evolution. The loss of mt-tRNA genes may be associated with selection pressure or transfer to the nucleus [69]. The numbers and sizes of introns in the 8 species of *Cladonia* were measured and compared, the size of introns from 2,617 to 13,642 which showed a big difference. In these species, the largest values were obtained in *Cladonia rangiferina* which contained 17 introns with a total length of 13,642 bp while the smallest values were obtained in *Cladonia peziziformis* which contained 4 introns with a total size of 2,617 bp.

**Fig 4:**
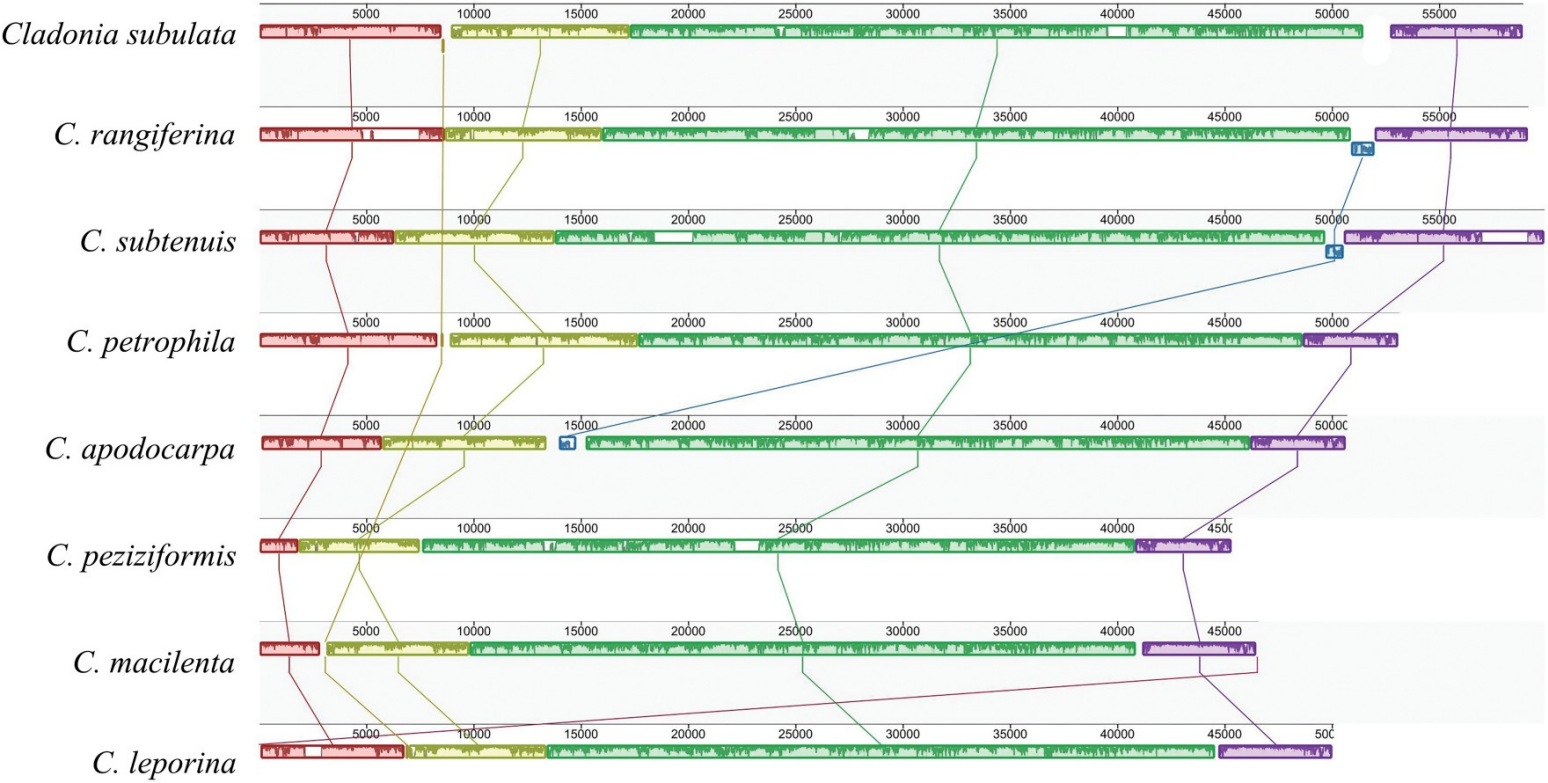
Collinearity analysis of eight mitogenomes from *Cladonia*. Homologous regions between different mitogenomes are represented by blocks of the same colour linked by lines. Note: The *C*. *subulata* mitogenome was adjusted beginning with the *cox*1 gene.

The synteny results obtained from in Mauve showed that the homologous collinear blocks occurred in all 8 fungal mitogenomes of *Cladonia* (Fig 4). The PCGs and rRNAs of the 8 species were obviously highly similar in composition. The 15 PCGs of these 8 species were *cox*1 -*nad*1 -*nad*4 -*rps*3 -*cob* -*cox*2 -*atp*9 -*atp*6 -*atp*8 -*nad*6 -*cox*3 -*nad*2 -*nad*3 -*nad*4L -*nad*5, and all genes were arranged in the same order in these 8 *Cladonia* species (S7 Table). At the synteny level, the mitogenome of *Cladonia subulata* showed a conserved in the gene arrangement and gene order of 15 PCGs, 2 rRNA genes and the majority of tRNA genes relative to those generally observed in *Cladonia*. Subsequently, in the mitochondrial genome of *Cladonia subulata*, it is divided into 6 gene clusters (Fig 4). The locations of the gene clusters were basically the same, but there were some differences in size. This main difference was located near *cox*1, which may be due to the length differences among homologous clusters caused by the acquisition or deletion of introns, resulting in a change in mitochondrial genome length (Fig 4). To better understand whether the change in homologous clusters was highly variable in litmus, 18 public complete mitochondrial genome sequences (including unannotated sequences) of *Cladonia* in NCBI were selected for further collinearity analysis. A total of 13 homologues were clearly identified the 18 mitochondrial genomes (S3 Fig). It was found not only that the mitochondrial genome of *Cladonia* showed a rearrangement phenomenon, but also that some homologous clusters were lost when more diverse samples were analysed. Interestingly, only *Cladonia uncialis* showed notable rearrangement and loss of homologous clusters, with four inverted and two lost homologous clusters. In a previous study, *Cladonia uncialis* showed inversion blocks of four genes (*nad*6, *cox*3, *nad*2 and *cox*3) compared with *C*. *rangiferina* [26]; however, combined with the results of gene arrangement and collinearity, there was no large-scale rearrangement in the other 17 *Cladonia* species which may indicate that the mitogenome of *Cladonia* is relatively conservated.

**Fig 5:**
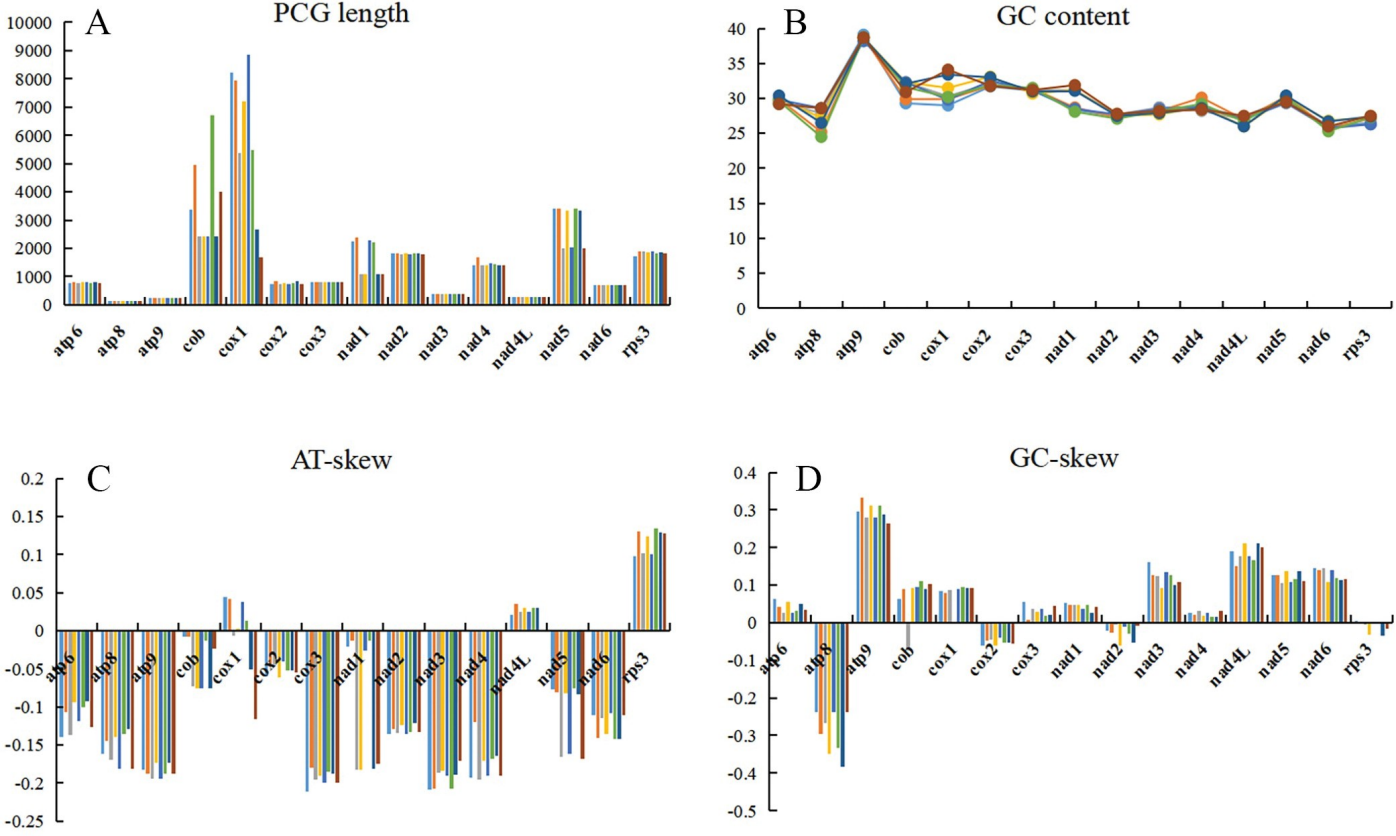
Variation in the length and base composition of each of 15 protein-coding genes (PCGs) in 8 mitochondrial genomes in the order *Cladonia*. A: PCG length variation; B: GC content; C: AT-skew; D: GC-skew. Different species are indicated by different colours.

A more in-depth comparative study of these 8 species is shown in Fig 5. Among the 15 PCGs, the lengths of *atp*8, *atp*9, *cox*3, and *nad*3 remained the same, while the lengths of other genes showed varying degrees of divergence (Fig 5A). The gene with the greatest length difference was *cox*1, which was related to the abundant introns it contained. The GC content in the *Cladonia subulata* mitogenome was similar to that in other species, below 40%. *atp*9 contained the highest GC content among all investigated species (Fig 5B). There was a negative AT skew in *atp*6, *atp*8, *atp*9, *cob*, *cox*2, *cox*3, *nad*1, *nad*2, *nad*3, *nad*4, *nad*5, and *nad*6, while *nad*4L and *rps*3 had a positive A-T skew. In addition, *cox*1 showed differences result in different species: *Cladonia subulata*, *C*. *rangiferina*, *C*. *leporina*, *C*. *petrophila*, and *C*. *subtenuis* were presented a positive A-T skew, but *C*. *apodocarpa*, *C*. *macilenta*, and *C*. *peziziformis* exhibited a negative A-T skew (Fig 5C).

Most PCGs had a positive G-C skew, while *atp*8, *cox*2 and *nad*2 showed a negative G-C skew. In addition, the G-C skew of *rps*3 in *Cladonia rangiferina* was zero which meant that the G and C were present in equal amounts, and only *rps*3 of *C*. *subulata* exhibited a positive G-C skew (Fig 5D).

The comparative analysis of tRNAs in *Cladonia* showed that *C*. *subulata* had two copies of *trn*L (Leu) and *trn*S (Ser), three copies of *trn*M (Met) and *trn*R (Arg) (S7 Table). Different species presented different tRNA copy numbers. *Cladonia peziziform* had three copies of *trn*M, and two copies of *trn*R, *trn*S, and *trn*L; *Cladonia macilenta* had no copies; *Cladonia leporina* had four copies of *trn*T, and three copies of *trn*M, and two copies of *trn*R, *trn*S, and *trn*L; *Cladonia apodocarpa* had three copies of *trn*M, and two copies of *trn*R, *trn*L, and *trn*K; *Cladonia petrophila* had two copies of *trn*M, *trn*R, *trn*S, and *trn*K; *Cladonia rangiferina* had three copies of *trn*R and two copies of *trn*L; and *Cladonia subtenuis* had three copies of *trn*N and *trn*R, and two copies of *trn*S and *trn*L (S4 Fig, S7 Table). The varying copy numbers in these species indicated that *Cladonia macilenta* may have lost at least one copy of *trn*M, *trn*R, *trn*S, *trn*L, *trn*T, and *trn*K or that duplications of these tRNAs have occurred in other species. Subsequently, *Cladonia subulata* may have lost one copy of *trn*K and three copies of *trn*T, or these two tRNAs may have been duplicated in other species. In general, sequences different in tRNA genes were connected with variation.

### Repeat sequences

**Fig 6:**
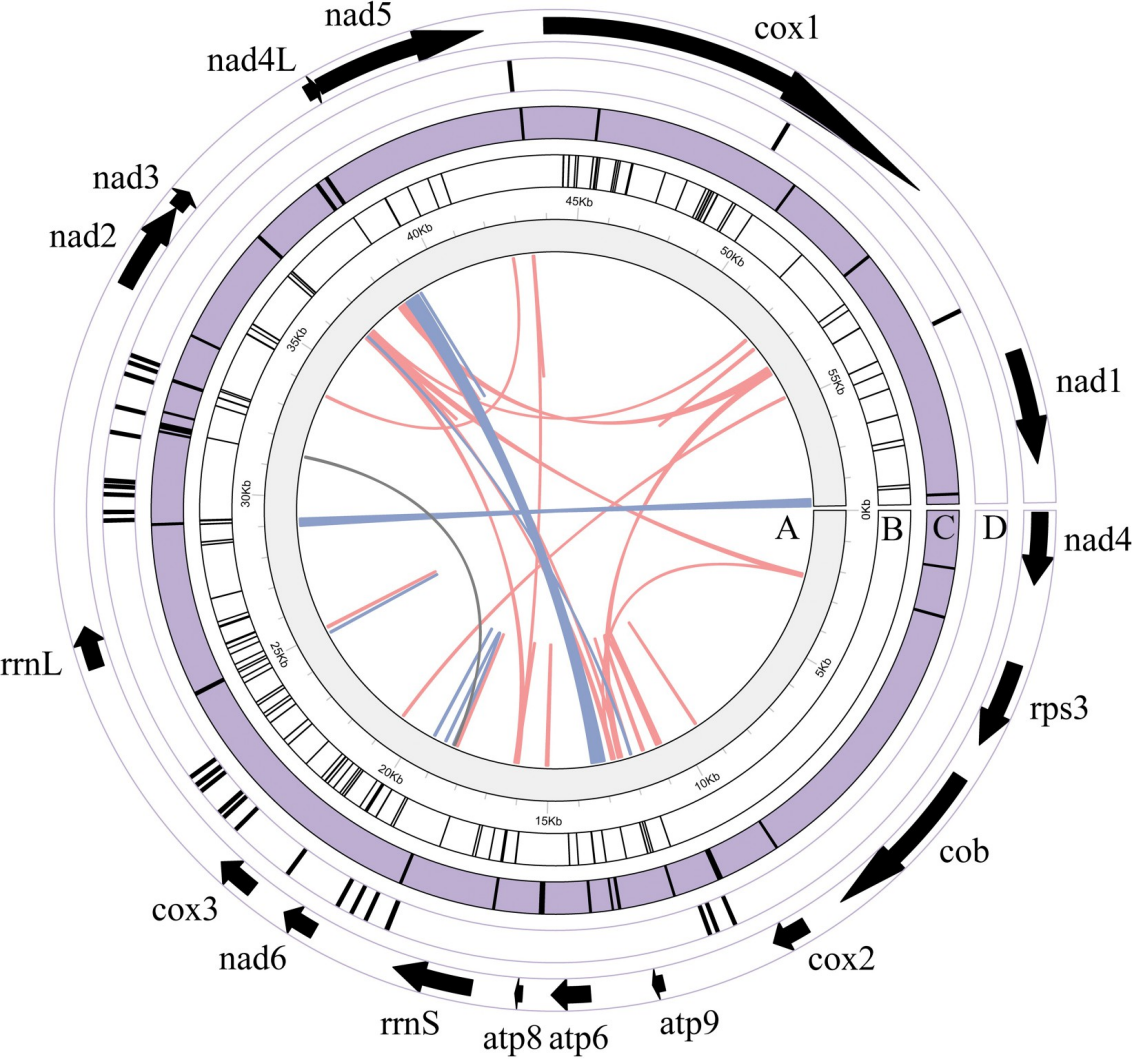
Location of repeats in *Cladonia subulata*. A: Detected by REUPuter; B: Detected by Tandem Repeat Finder; C: Detected by MISA; D: The tRNA location. The PCGs, *rrnS* and *rrnL* and their length in *C*. *subulata* are shown by arrows, and the direction is the direction of translation. Orange lines indicated forward repeats, blue lines indicated palindromic repeats, and grey lines indicated reverse repeats. The circos map was drawn by TBtools [72].

BiBiserv-REPuter can locate and identify forwards, reverse, complementary and palindromic repeat sequences [52]. The accumulation of repetitive sequences in the fungal mitogenome over time may lead to dynamic changes in genome structure, and in turn affect the rearrangement of the mitogenome [70]. In the mitogenome of *Cladonia subulata*, REPuter identified 67 forward types (totalling 2,640 bp), 22 palindromic repeats (totalling 1,456 bp) and 1 reverse repeat of only 30 bp (S8 Table). Tandem repeats were searched by using Tandem Repeats Finder, which identified 30 tandem repeats in *Cladonia subulta*, and the longest tandem sequence of *C*. *subulata* was 42 bp. Most tandem repeats were located in the intergenic spacer, and some were located in the *cox*1 gene (Fig 6, S9 Table). Simple sequence repeats (SSRs) are a valuable type of molecular marker that may show high variation within a given species and has been used in population genetics and polymorphism research [71]. We analysed the occurrence and types of SSRs in the mitochondrial genome of stamens, and a total of 93 SSRs were identified (Tables 3 and S10). Most of these SSRs were composed of single-nucleotide and double-nucleotide repeats, which were found 36 times and 50 times, respectively, and trinucleotide repeats (2), tetranucleotide repeats (3), and hexanucleotide repeats (2) were also present at low frequencies. All single-nucleotide repeats consisted of A/T repeats. Similarly, all dinucleotide repeats consisted of AT/AT repeats (Tables 3 and S10). The results showed that the mitochondrial genome SSRs were mainly composed of short PolA or PolyT repeats and rarely contained tandem G or C repeats. It can be seen from Fig 6 that most of the repetitions were located in the gene intervals.

**Table 3:** Numbers of SSRs identified in the mitogenome of *Cladonia subulata*.

Repeats	3	4	5	6	7	8	9	10	11	12	total
**A/T**	/	/	/	/	/	20	8	5	2	1	36
**AT/AT**	/	34	11	5	/	/	/	/	/	/	50
**AAC/GTT**	/	1	/	/	/	/	/	/	/	/	1
**AAT/ATT**	/	1	/	/	/	/	/	/	/	/	1
**AAAT/ATTT**	1	/	/	/	/	/	/	/	/	/	1
**AAGC/CTTG**	1	/	/	/	/	/	/	/	/	/	1
**ACAT/ATGT**	1	/	/	/	/	/	/	/	/	/	1
**AAAAAT/ATTTTT**	1	/	/	/	/	/	/	/	/	/	1
**AAACAT/ATGTTT**	1	/	/	/	/	/	/	/	/	/	1

### Phylogenetic analysis

**Fig 7:**
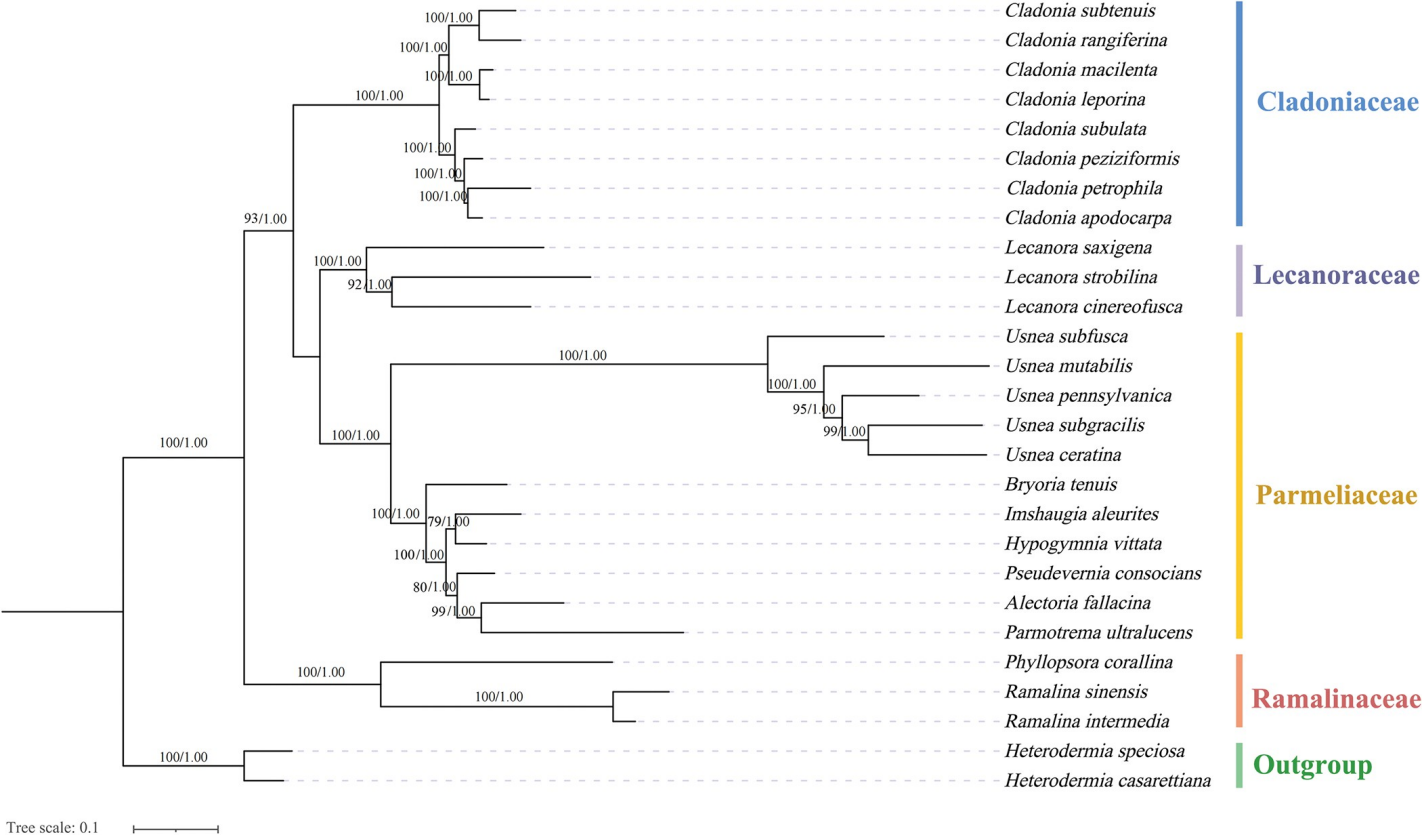
Phylogeny of Lecanorales based on the 23 available mitochondrial genomes based on the Bayesian inference (BI) and maximum likelihood (ML). Numbers on branches indicate posterior probability (BI) and bootstrap support (ML). *Cladonia* is framed with a dotted blue box. The species and GenBank accession numbers of the mitogenomes used in the phylogenetic analysis are provided in S3 Table.

The two phylogenetic trees (maximum likelihood tree and Bayesian inference tree) lead to the same topology with different supporting value. Phylogenetic analysis based on 23 mitochondrial genome PCGs of Lecanorales revealed division into four clades with high support (Fig 7). The phylogenetic analysis showed that there was a closer genetic relationship between Parmeliaceae and Lecanoraceae. All the *Cladonia* species were well clustered in the monophyletic branch of the Cladoniaceae. The results were similar to the previous multigene Lecanoromycetes phylogenetic analysis [58,59]; however, these researches also indicated that a certain portion of the phylogeny was less stable between Parmeliaceae and Lecanoraceae due to a higher concentration of taxa with only two (RNA-coding) genes, which suggests that more sequence loci were beneficial for constructing a stable phylogenetic tree.

**Fig 8:**
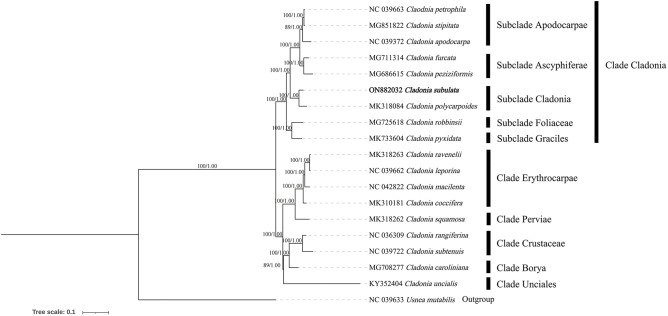
Phylogenetic analysis of eighteen complete mitochondrial genome in *Cladonia*. ModelFinder was used to select the best-fit model using AIC criterion. Best-fit model of ML tree according to AIC: GTR+F+R4. Best-fit model of BI tree according to AIC: GTR+F+G4. (ML/BI).

Finally, to obtain a better-supported phylogenetic tree of *Cladonia*, phylogenetic analysis was carried out on the complete mitogenomes of 18 *Cladonia subulata* (including annotated and nonannotated mitogenomes), *Usnea mutabilis* as the outgroup; and the conditions for performing the phylogenetic analysis were the same as those applied for Lecanorales (Fig 8). Our phylogenetic results were similar to the most recent multiple-locus (nuclear and protein loci) phylogeny of the genus *Cladonia* and some previous studies [73–76]. According to the results (Fig 8), *Cladonia subulata* formed a well-supported clade with *C*. *polycarpoides*, both species have “non-classic” cup-shaped. *Cladonia furcata* formed a high supported clade with *C*. *peziziformis*, which belonged to the subclade *Ascyphiferae* according to they contained the fumarprotocetraric acid and sparsely or moderately branched. Both our results and Brigham’s [75] showed that the *Cladonia stipitata* and *C*. *petrophila* had a more related relationship than *C*. *apodocarpa* in subclade *Apodocarpae*, most of them with non-calcareous substrates, ascending squamules, rare podetia, fumarprotocetraric acid present. Interestingly, Lendemer’s [74] study showed that *Cladonia stipitata* were morphologically similar to *C*. *petrophila* but *C*. *stipitata* formed a well-supported monophyletic group which distinct from *C*. *apodocarpa* and *C*. *petrophila* in phylogenetic results, the difference between us requires more mitogenomes of these three species for further phylogenetic analysis to get a more accurate phylogenetic result. In addition, *Cladonia robbinsii* and *C*. *pyxidata* belong to the subclade *Foliaceae* and subclade *Graciles* respectively. The subclade *Cladonia*, subclade *Ascyphiferae*, subclade *Apodocarpae*, subclade *Foliaceae* and subclade *Graciles* belong to Clade *Cladonia*. *Cladonia leporina* and *C*. *ravenlii* belonged to the Clade *Erythrocarpae* along with *C*. *coccifera* and *C*. *macilenta* [33,76] according to their red hymenial disc. *Cladonia subtenuis* and *C*. *rangiferina* formed a well-supported clade which belonged to Clade *Crustaceae*, the species in this clade were widely distributed in arctic and boreal to temperate areas. *Cladonia uncialis*, *C*. *caroliniana* and *C*. *squamosa* belong the Clade *Unciales*, Clade *Borya*, Clade *Perviae* according to the Stenroos’s [73] results. In addition, all clades within the tree had sufficient bootstrap support (Fig 8), it was showed the Clade *Erythrocarpae* had related relationship with Clade *Pervia*, and the Clade *Unciales*, Clade *Borya* and Clade *Perviae* showed a more related relationship than other Clades. Over all, the PCGs showed a great application in *Cladonia* phylogenetic analysis.

The sequencing of mitochondrial genes has been widely performed in fungal studies [77,78]. In lichenized fungi, although high variability of ITS sequences was found in the studies, SSU, LSU and *cox* genes have been widely used in taxonomic studies of lichenized fungi and have shown greater stability when used in phylogenetic construction in previous studies [79–82].

## Conclusion

To our knowledge, this study was the first in which the mitogenome of *Cladonia subulata* was sequenced and assembled, and its overall characteristics were annotated and analysed, its mitogenome main characteristics were similar to those observed in other species of the genus. The *cox*1 gene showed the largest variation among all PCGs because of abundant introns. The presence/absence of introns indicated that introns were related to the evolutionary dynamics of mitotic introns [6]. Subsequently, the duplication of the tRNAs of the *C*. *subulata* mitogenome may have resulted in a number of repeat/loss events during evolution.

Comparative analysis of mitogenome alignment and structure can be used to reveal evolutionary relationships among species [83]. In the gene collinearity analysis indicated dynamic changes in the mitogenome of *Cladonia*. *Cladonia uncialis* showed a larger change in homologous clusters than the other species, giving rise to the question of whether this phenomenon is common or specific to *Cladonia*. Further statistical analysis will be required to answer this question. The repeat sequences of *Cladonia subulata* mitogenome were mainly distributed in gene intervals (Fig 6), which are more likely to show variation than coding sequences. The vast majority of sequences were composed of A/T nucleotides, which was consistent with the high A/T content (70.70%) of the genome. Therefore, whether a high A/T content is related to high variation is worthy of further studying.

This study provides new insights into the genetics of *Cladonia* and comparative analysis of eight fungal mitogenomes provides an understanding of the mitochondrial evolution in the genus, which will provide data support for further study and also protect the diversity of *Cladonia* genes to some extent.

## Supporting information

S1 Fig Photo of *Cladonia subulata*. From Urumqi No.1 Glacier, Tianshan Mountains of Xinjiang, China (43°13′30″ N, 87°9′11″ E).
(DOCX)

S2 Fig ITS, SSU and *RPB*2 concatenated phylogenetic three of *Cladonia* by MrBayes. The *Cladonia subulata* were included in the same clade with a high support. Sequences obtained in this paper are **bolded**.
(DOCX)

S3 Fig Collinearity analysis of 18 *Cladonia* mitochondrial genomes. 13 homologous were detected among the 18 mitochondrial genomes. The size and relative positions of homologous regions varied across mitochondrial genomes.
(DOCX)

S4 Fig Copies of tRNAs in *Cladonia*. (DOCX)

S1 Table The condition of PCR. (XLSX)

S2 Table ITS and SSU NCBI numbers were been used in paper. (XLSX)

S3 Table The mitogenome of Lecanorales NCBI numbers. (XLSX)

S4 Table The best fit model for Maximum likelihood and Bayesian analyses of Lecanorales. (XLSX)

S5 Table The mitogenomic annotation details of *C*. *subulate*. (XLSX)

S6 Table Codon usage analysis of *C*. *subulate*. (XLSX)

S7 Table Gene order of the *Cladonia* mitogenomes. (XLSX)

S8 Table Repeat loci in the mitogenome of *C*.*subulata* as revealed by REPuter. (XLSX)

S9 Table Tandem repeats detected in the mitogenomes of *C*. *subulate*. (XLSX)

S10 Table Microsatellite DNA in *C*. *subulate*. (XLSX)
