# Supplementary material for: De novo mitochondrial genome sequencing of Cladonia subulata and phylogenetic analysis with other dissimilar species
Source: PLoS One. 2023 May 23;18(5):e0285818. doi: 10.1371/journal.pone.0285818 (PMC10204972; doi:10.1371/journal.pone.0285818)
Supplement: S1 Fig — From Urumqi No.1 Glacier, Tianshan Mountains of Xinjiang, China (43°13′30″ N, 87°9′11″ E). (DOCX) [file pone.0285818.s001.docx]

**Characterization and phylogenetic study of the Cladonia subulata (Cladoniaceae, Ascomycota) mitochondrial genome, and comparison with dissimilar species**

Jinjin Fang^1^, Lidan Wang^1^, Gulmira Anwar^1^, Reyim Mamut^1^*

1College of Life Science and Technology, Xinjiang University, Xinjiang 830046, Urumqi, China,

***Corresponding author:**[**reyim_mamut@xju.edu.cn**](mailto:reyim_mamut@xju.edu.cn)

**Supplementary figures**


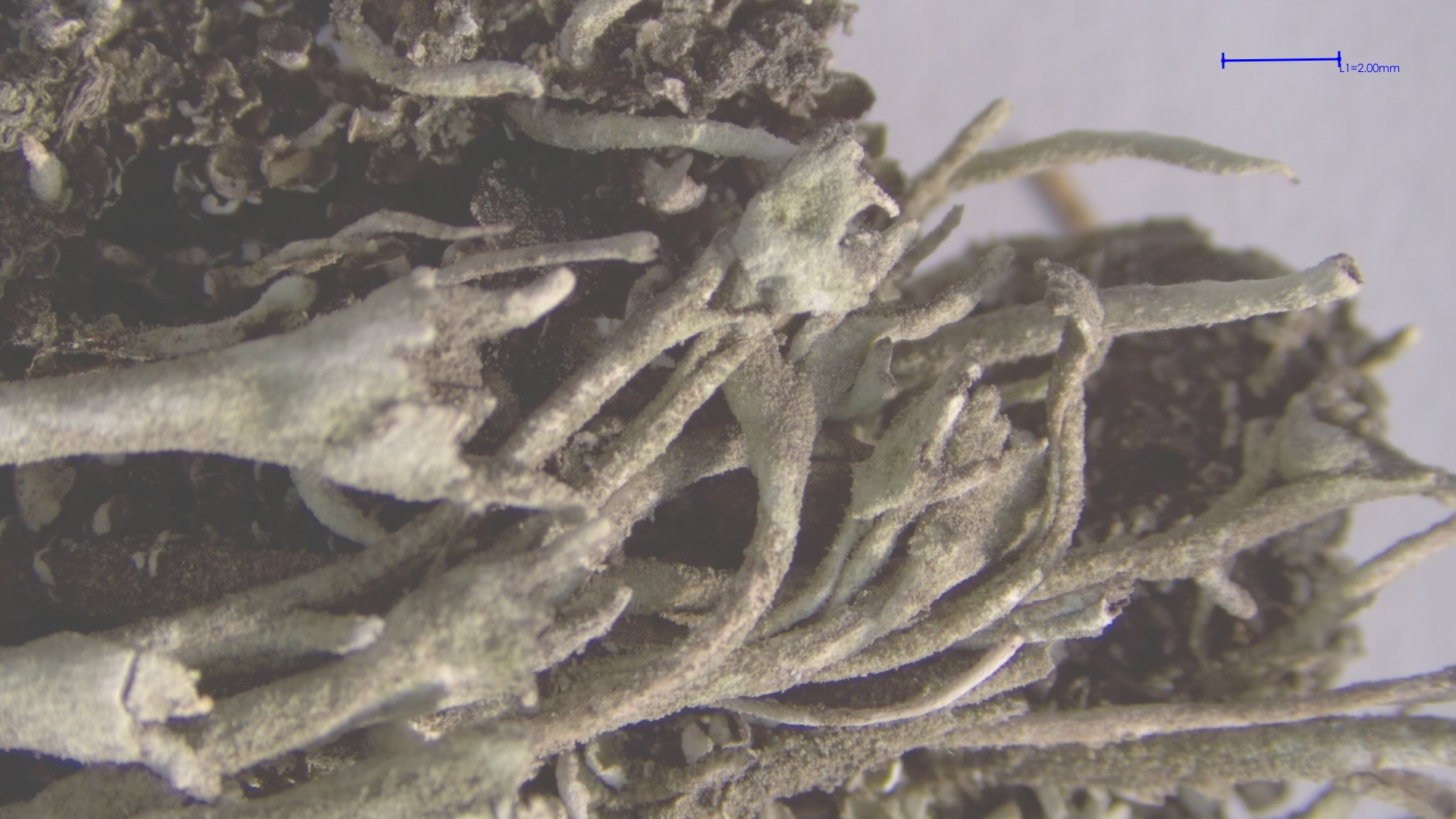


**Fig S1. Photo of *Cladonia subulata*.** From Urumqi No.1 Glacier, Tianshan Mountains of Xinjiang, China (43°13′30″ N, 87°9′11″ E)
