# Supplementary material for: De novo mitochondrial genome sequencing of Cladonia subulata and phylogenetic analysis with other dissimilar species
Source: PLoS One. 2023 May 23;18(5):e0285818. doi: 10.1371/journal.pone.0285818 (PMC10204972; doi:10.1371/journal.pone.0285818)
Supplement: S2 Fig — The Cladonia subulata were included in the same clade with a high support. Sequences obtained in this paper are bolded. (DOCX) [file pone.0285818.s002.docx]

**Fig S2. ITS, SSU and *RPB*2 concatenated phylogenetic three of *Cladonia* by MrBayes.** The *Cladonia subulata* were included in the same clade with a high support. Sequences obtained in this paper are **bolded**.
