# Supplementary material for: De novo mitochondrial genome sequencing of Cladonia subulata and phylogenetic analysis with other dissimilar species
Source: PLoS One. 2023 May 23;18(5):e0285818. doi: 10.1371/journal.pone.0285818 (PMC10204972; doi:10.1371/journal.pone.0285818)
Supplement: S3 Fig — 13 homologous were detected among the 18 mitochondrial genomes. The size and relative positions of homologous regions varied across mitochondrial genomes. (DOCX) [file pone.0285818.s003.docx]

**Fig S3. Collinearity analysis of 18 *Cladonia* mitochondrial genomes.** 13 homologous were detected among the 18 mitochondrial genomes. The size and relative positions of homologous regions varied across mitochondrial genomes.
