# Supplementary material for: De novo mitochondrial genome sequencing of Cladonia subulata and phylogenetic analysis with other dissimilar species
Source: PLoS One. 2023 May 23;18(5):e0285818. doi: 10.1371/journal.pone.0285818 (PMC10204972; doi:10.1371/journal.pone.0285818)
Supplement: S4 Fig — (DOCX) [file pone.0285818.s004.docx]

**Fig S4. Copies of tRNAs in *Cladonia*.**
